# Poor Air Quality Is Linked to Stress in Honeybees and Can Be Compounded by the Presence of Disease

**DOI:** 10.3390/insects14080689

**Published:** 2023-08-04

**Authors:** Christopher Mayack, Sarah E. Cook, Bernardo D. Niño, Laura Rivera, Elina L. Niño, Arathi Seshadri

**Affiliations:** 1 USDA/ARS/WRRC, Invasive Species and Pollinator Health Research Unit, Davis, CA 95616, USA; christopher.mayack@usda.gov (C.M.); bdnino@ucdavis.edu (B.D.N.); lwrivera@ucdavis.edu (L.R.); 2 SpecialtyVETPATH, 3450 16th Ave. W. Ste 303, Seattle, WA 98119, USA; sestevens@ucdavis.edu; 3 Department of Pathology, Microbiology, and Immunology, School of Veterinary Medicine, University of California, 944 Garrod Drive, Davis, CA 95616, USA; 4 Department of Entomology and Nematology, University of California, 1 Shields Avenue, Davis, CA 95616, USA; elnino@ucdavis.edu

**Keywords:** air quality index, *Apis mellifera*, HSP70, *Nosema ceranae*, temperature stress, vitellogenin

## Abstract

**Simple Summary:**

Climate change is associated with warmer and drier weather on average in central California. At the same time, honeybees are being transported from all over the United States to California for the completion of pollination services that ensure increased yields for a variety of crops, resulting in bees experiencing ambient abiotic stressors. Higher temperatures can reduce air quality, further exacerbating honeybee health challenges. We investigated the relationship between higher daily temperatures and poor air quality and demonstrated associations with incidences of pests (e.g., *Varroa* mites) and pathogens (e.g., *Nosema ceranae*). We also correlated the expression of genes linked to immune functioning, oxidative stress, and buffering against temperature and air quality stressors. High daily temperatures are associated with poorer air quality, and they are both associated with lowered immune system functions and increased oxidative stress protection against pests and diseases. Higher *Varroa* mite loads are correlated with the lower potential ability of honey bees to buffer against temperature stress. Our study provides insights into interactions between climate change-related abiotic stressors and their relation to biotic stressors, which underlie a decline in honeybee health.

**Abstract:**

Climate change-related extreme weather events have manifested in the western United States as warmer and drier conditions with an increased risk of wildfires. Honeybees, essential for crop pollination in California, are at the center of these extreme weather events. We associated the maximum daily temperature and air quality index values with the performance of colonies placed in wildfire-prone areas and determined the impact of these abiotic stressors on gene expression and histopathology. Our results indicate that poor air quality was associated with higher maximum daily temperatures and a lower gene expression level of Prophenoloxidase (*ProPO*), which is tied to immune system strength; however, a higher gene expression level of Vitellogenin (*Vg*) is tied to oxidative stress. There was a positive relationship between *Varroa* mites and *N. ceranae* pathogen loads, and a negative correlation between *Varroa* mites and Heat Shock Protein 70 (*HSP70*) gene expression, suggesting the limited ability of mite-infested colonies to buffer against extreme temperatures. Histological analyses did not reveal overt signs of interaction between pathology and abiotic stressors, but *N. ceranae* infections were evident. Our study provides insights into interactions between abiotic stressors, their relation to common biotic stressors, and the expression of genes related to immunity and oxidative stress in bees.

## 1. Introduction

Ongoing changes to climatic conditions are resulting in frequent extreme temperature fluctuations and a rise in weather-related natural disasters, which, in turn, affect ecosystems, and the species within [1]. Wildfires, temperature extremes, drought, and flooding in California, are now more prevalent than in the past and are predicted to worsen in the future [2]. In general, climate change is expected to affect global health, distribution, and the abundance of organisms [3,4,5], and pollinators are no exception to this [6,7]. The western honeybee (*Apis mellifera*) is crucial for pollination in natural and agricultural ecosystems, with annual economic contributions estimated to be over $200 billion globally [8,9] and over $18 billion in the United States [10,11]. In recent years, there has been an ongoing decline in bee health attributed mainly to pests and pathogens, poor nutrition, and pesticide exposure [12,13,14,15,16], and bees are facing further challenges in relation to rapidly changing weather patterns. A global decline in pollinator biodiversity has at least in part been attributed to the effects of climate change [7,17]. For climates like California, which are amenable for agriculture production, climate-change-mediated warmer temperatures impact insect pollinators and, thus, their pollination efficiency, eventually affecting agricultural production [18]. Beekeepers have raised concerns and reported adverse consequences related to severe weather events, including the loss of colonies to wildfires, weakened colonies from a scarcity of nectar and pollen due to changing phenology and bloom periods [19,20], decreased honey production, and greater pathogen loads exacerbating ongoing challenges to pollinator health [21,22].

Another direct consequence of extreme weather events [23] in relation to climate change is poor air quality which can have a direct negative impact on honeybee health [24,25]. Honeybees have previously been used as sentinels to monitor environmental pollution [26,27], but little attention has been given to the detrimental impacts of pollution on bees themselves. As beekeepers from across the United States bring their colonies to California for crop pollination, honeybee colonies are substantially exposed to the adversities of climate-related extremes in California, even if only for part of the year [28,29]. In addition, most queen honeybees and colonies in the rest of the country come from California, requiring the beekeeping and queen-rearing industries to maintain robust colonies throughout the year. While climate change by itself is likely a substantial stressor on honeybee health, its interactions with other existing biotic stressors can further compound these impacts, though, at this time, the nature of these interactions is not fully clear.

Of the several biotic stressors that affect honeybees, parasitic mites, such as *Varroa destructor,* affect brood and adult bees, and chronic infections from the pathogen, *Nosema ceranae,* affect adult bees and are critically important. The challenges from these biotic stressors may be compounded as bees adapt to abiotic stressors resulting from changing weather patterns. Our goal in this study is to assess the relationship between two main abiotic factors—temperature and air quality—and explore their relation to the two above-mentioned biotic stressors. Specifically, we investigated how daily maximum temperatures and poor air quality relate to each other and to *N. ceranae* and *V. destructor:* the chronic biotic stressors. While *N. ceranae* is relatively less virulent, it suppresses the immune system and causes energetic stress and metabolic dysregulation that could potentially be compounded by other stressors [12,16,30,31]. *Varroa* mites, on the other hand, are central to declines in bee health as they nutritionally deplete the honeybee by directly feeding on them [32] and vector more virulent strains of viruses [33].

We then assayed the gene expression of stress biomarkers that are activated in response to different stress pathways with the goal of gaining mechanistic insight into the interactions between abiotic and biotic stressors as they impact honeybee health. We measured the gene expression for *Heat Shock Protein 70* (*HSP70*), a general marker for the ability of bees to buffer against stress from extreme temperature changes [34], *Prophenoloxidase* (*ProPO*), a marker for immune upregulation in response to disease stress [35] and *Vitellogenin* (*Vg*), a general physiological stress marker known to be a master regulator with ties to oxidative stress, immune functioning, development, and metabolism [36]. We further assayed individual bees for morphological signs (histologic imaging) of stress and for pathologic lesions of *N. ceranae* incidence as secondary measures of the potential adverse impacts of temperature and poor air quality. Histologic imaging, which involves the microscopic evaluation of tissues and cells, has more recently gained the attention of honeybee researchers [37,38,39,40,41,42].

## 2. Materials and Methods

### 2.1. Sample and Environmental Data Collection

**Figure 1:**
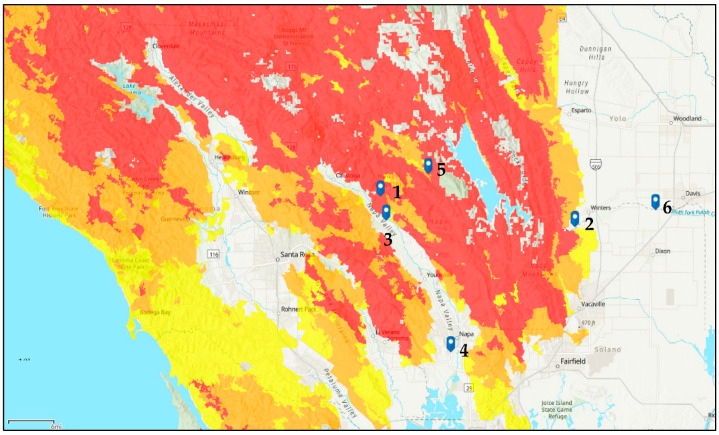
Six study sites and their Fire Hazard Severity Zones as defined by the California Department of Forestry and Fire Protection. (CAL FIRE; https://osfm.fire.ca.gov/divisions/community-wildfire-preparedness-and-mitigation/wildfire-preparedness/fire-hazard-severity-zones/, accessed on 4 April 2023). Fire hazard zones: 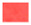 Very high 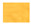 High 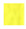 Moderate.

This study was conducted from June to November of 2021 with 12 standard Langstroth hives consisting of a total of 18 frames and a 5.68 L (1.5 gal) feeder. The hives were equalized in strength and were placed near areas that were known to be prone to wildfires, including six different locations in the Napa, Yolo, and Solano counties of central California (Figure 1). Hives in all the locations except in sites 3 and 4 (treatment-free operations) received recommended best hive practices [43,44]. All the hives were fed weekly with 3.79 L (1 gal) of a pro-sweet liquid feed (Mann Lake Ltd., Mann Lake) and ultra-bee pollen patty (Mann Lake Ltd., Mann Lake). As they returned from foraging trips, approximately 150 forager bees were collected from each colony at the entrance to the hive using an insect vacuum and were then placed in 50 mL Falcon tubes labeled by the date, hive number, and location. A total of approximately 150 in-hive bees were collected on the same day by rolling bees down a central capped brood frame of the hive into a separate 50 mL Falcon tube. Monthly collections were completed, samples were stored on dry ice after field collections and then transferred to a −80 °C freezer until further analyses. From the weather station closest to each apiary location, historical maximum daily temperatures for each sampling date were retrieved from the Farmer’s Almanac (https://www.farmersalmanac.com/long-range-weather-forecast, accessed on 15 February 2023) and the air quality index (AQI) for each sampling date was noted from AirNow.gov (https://www.airnow.gov/, accessed on 15 February 2023).

### 2.2. Measuring Colony Performance Metrics

The monthly, detailed assessments included recording the Frames of Bees (FOB), the presence of a laying queen as indicated by the presence of eggs, and measuring the amount of brood, pollen, nectar, honey, and the amount of empty space on a frame, on a scale of 0–10. *Varroa* mite loads for each colony were also measured via the standard alcohol wash method [45]. Approximately 300 bees were scooped with a cup from a brood frame from each hive to estimate the total number of *Varroa* mites in the hive. Colonies with mite levels at over 3% of the threshold received mite control treatments [46]. The frames of bees (FOB) measure is an effective method to quickly estimate the population size of adult bees in colonies. This was completed by counting the top and bottom of frames covered with bees in the hive. In-hive bees, not on the central part of the frames but present on the top of frames and at the bottom of the hive box, were estimated as one more FOB and were added to the total for each hive, providing an estimate of the adult bee strength. By contrast, the number of adult bees was assessed by measuring how much area the bees occupied on the side of each frame in the hive.

### 2.3. Sample Preparation for Molecular Analysis

In-hive and forager bees were placed in separate 50 mL falcon tubes and were thawed, macerated, and homogenized in 5 mL of nuclease-free water (VWR, Radnor, PA, USA) per 30 collected bees, using a 50 mL disposable tissue grinder (VWR, Radnor, PA, USA). The bee homogenate was then aliquoted as a 200 µL volume into three different 1.5 mL microcentrifuge tubes, which were stored in a −80 °C freezer until further analysis.

### 2.4. Nosema qPCR Quantification

DNA extraction to quantify *Nosema* infection loads was adapted from [12]. Briefly, 300 µL of the HBRC buffer was added to 200 μL of bee homogenate, and this was treated with 4 µL (20 mg/mL) of proteinase K for 3 h at 60 °C. A total of 300 µL of 1:1 phenol-chloroform was added, and this was centrifuged at 13,000 rpm for 5 min. This step was repeated once, and the supernatant was then added to a 1.5 mL microcentrifuge tube that contained 300 μL of chloroform. This was then centrifuged at 13,000 rpm for 5 min. Finally, the supernatant was added to 30 μL of a 3 M sodium acetate (pH of 5.2) along with 600 μL of 95% ethanol to precipitate the DNA overnight at −20 °C. The amount and purity of the extracted DNA were assessed using a Thermo Fisher Scientific NanoDrop spectrophotometer (Waltham, MA, USA). Each DNA extraction was diluted to 10 ng/μL for the qPCR template DNA. Methods for the quantification of *Nosema* and the identification of the species from a melt curve analysis were performed following [47]. The 25 μL duplex qPCR reaction consisted of 0.4 uL of ROX (reference dye), 12.5 μL of 2x GoTaq Promega qPCR master mix, a 175 nM F-NAPIS primer (0.4375 μL), a 175 nM R-NAPIS primer (0.4375 μL), 350 nM F-NCERANAE (0.875 ul), 350 nM R-NCERANAE (0.875 μL) (Table S1), 1 μL of template DNA, and 8.475 μL of nuclease-free water. The thermocycler settings were as follows: a 10 min denaturing period at 95 °C, 40 cycles of 30 s denaturing at 95 °C, 30 s annealing at 60 °C, 30 s extension at 72 °C, and a 5 min extension period at 72 °C. The melt curve analysis was as follows: 1 min at 95 °C and 30 s at 55 °C with 40 successive 30-s increases of 1 °C. Each run contained *N. apis* and *N. ceranae* positive control as well as a negative control. The samples were run in the Biorad CFX384 qPCR thermocycler (Hercules, CA, USA) in technical duplicates. If the standard deviation between duplicates was greater than one, the samples were re-run. The amount of *Nosema* DNA that was quantified was converted to the total number of *Nosema* copies using the standard curves provided [47]. 

### 2.5. Gene Expression Analysis

All primer set sequence information used for gene expression analysis can be found in Table S1. Methods for the RNA extraction of other gene expression analyses were adapted from [48]. Briefly, a 5 mm steel bead was added to 200 µL of the bee homogenate in a 2 mL Eppendorf safe-lock microcentrifuge tube. The top of the tube was wrapped in parafilm, and the tube was placed in the BeadBlaster™ 24 (Benchmark Scientific, Sayreville, NJ, USA) and was set to 4:00 m/s for 1 cycle with a 15 s interval, and the duration of the run time was 30 s. A 1% β-mercaptoethanol RLT buffer solution was added to the samples; the RLT buffer solution was part of the Qiagen RNeasy Plant Mini Kit (Germantown, MD, USA). The mixture was vortexed for 30 s. Then, the RNA extraction proceeded by following the manufacturer’s instructions of the Qiagen RNeasy Plant Mini Kit (Germantown, MD, USA) with an Invitrogen™ TURBO™ DNase kit (Waltham, MS, USA) on column treatment to remove any genomic DNA contamination. The amount and purity of the extracted RNA were assessed using a Thermo Fisher Scientific NanoDrop spectrophotometer (Waltham, MA, USA).

A blend of random hexamers and an oligo dT SensiFAST cDNA Synthesis Kit (Newtown, OH, USA) was used following the manufacturer’s instructions to generate cDNA from the extracted RNA standardized to 700 ng. A 20 µL cDNA synthesis reaction was carried out involving 4 µL of a 5x TransAmp Buffer, 1 µL of reverse transcriptase, and 15 µL of extracted RNA. The relative gene expression of a total of three targets, *Heat Shock Protein 70* (*HSP70*), *Vitellogenin* (*Vg*), and *Prophenoloxidase* (*ProPO*), were quantified relative to the reference gene *Ribosomal Protein S5* (*RpS5*). The following qPCR protocols were used for each target. For the quantification of the *Vg* and *RpS5* expression, the final volume of the reaction was 10 µL which consisted of 5 µL of qPCR 2x Promega (Madison, WI, USA) master mix (no ROX), 0.2 µL (10 µM) of either the Vg-F or Vg-R primers or the RpS5-F and RpS5-R primers, respectively, including 2 µL of template cDNA, and 2.6 µL of nuclease-free water. The qPCR thermocycler program was: 95 °C for 5 min, 45 cycles of 95 °C for 10 s, and 60 °C for 20 s, with a final extension step of 72 °C for 30 s [49]. For ProPO analysis, the following 20 μL of the qPCR reaction was used: 10 μL of 2x qPCR master mix, 1 μL (10 μM) of each F-ProPO and R-ProPO primer, 6.4 μL nuclease-free water, 0.4 μL of ROX Reference dye II, and 1 μL of template cDNA. The following qPCR thermocycler program was used for ProPO: 10 min at 95 °C, followed by 40 cycles of 20 s at 95 °C, 20 s at 55 °C, and a final extension step of 30 s at 72 °C [35]. For HSP70, the following 25 μL qPCR reaction was used: 12.5 μL of a 2x qPCR master mix (no ROX), 0.5 μL (10 µM) of the F-HSP70 primer, 0.5 μL of the R-HSP70 primer, 9.5 μL of nuclease-free water, and 2 μL of template cDNA. The qPCR thermocycler program consisted of: 95 °C for 15 min, 40 cycles at 95 °C for 20 s, 58 °C for 30 s, and a final extension step of 72 °C for 30 s [34]. Each run consisted of technical duplicates and was run in the Biorad CFX384 qPCR thermocycler (Hercules, CA, USA). If the standard deviation for the reference gene was greater than one, then the samples were re-run, and if for each target it was greater than two, the samples were re-run. The relative gene expression was calculated using the delta–delta Ct method.

### 2.6. Histology Methods

Methods were adapted from Cook et al. (In press). Briefly, honeybees were collected and stored in 10% neutral buffered formalin (NBF) and were fixed for a minimum of 24 h. The head, thorax, and abdomen were separated using spring scissors (World Precision Instruments, Sarasota, FL, USA) or a steel surgical blade (Miltex, NY, USA). The head and thorax were subsequently sectioned transversely, and the abdomen was sectioned in a sagittal orientation along the midline using a carbon steel surgical blade (Miltex, NY, USA). Blades were replaced with at least every three samples to prevent dulling, and all tissues were placed with their cut surface down in either one or two tissue cassettes (Tissue-Tek). All cassettes were stored in 10% NBF until further routine laboratory processing, including routine paraffin embedding, sectioning at 4 µM sections, mounting onto glass slides, and stained with hematoxylin and eosin (H&E). Two consecutive sections of all three body segments were mounted onto a single glass slide for light microscopic evaluation and subsequent image analysis. 

Based on the results of molecular analysis for gene expression, targeted groups were evaluated via light microscopy and image analysis. For microscopic evaluation, all body segments and tissues were assessed, and any lesions were documented. For image analysis, the total surface area for hypopharyngeal glands located dorsal to the central nervous system within the head section was calculated, as was the mandibular gland surface area. For mandibular glands, if both left and right glands were in the plane section of the slide, then both sides were measured and divided by two to obtain an average for one side. If only one side was in the section, then just that half was measured. The surface area of the glands was measured using Adobe Photoshop (v 24.1.1).

Finally, each bee was histologically evaluated for Nosemosis to compare the prevalence diagnosed histologically with the molecular quantification of *Nosema*. Bees were sampled monthly from June to November 2021. Six sites were sampled from June to September, and 10 nurse and 10 forager bees from each site were processed (a total of 120 bees for each time point). One site was discontinued in September as the colonies died, and the remaining five sites were sampled similarly in October and November (100 bees for each time point). 

### 2.7. Statistical Analyses

All statistical analyses were carried out using JMP Pro v. 16 (SAS Institute, Cary, NC, USA) or GraphPad Prism (San Diego, CA, USA). Data visualization was carried out using GraphPad Prism (San Diego, CA, USA). All numeric variables (*N. ceranae* load, air quality index, maximum daily temperature, the normalized gene expression of HSP70, ProPO, and Vg, FOB, *Varroa* mite counts, adult bees, the amount of the brood, the amount of honey, the amount of nectar, the amount of pollen, and the amount of empty space in the hive) did not follow a normal distribution. All count data were over-dispersed. Therefore, a generalized linear negative binomial regression model was used where the date of collection, the apiary sampled, and whether the bees collected were foragers or in-hive bees were included in the full model as random effects. The apiary, date, and age class of the bee had significant random effects when analyzing the gene expression of biomarkers for *N. ceranae* and *Varroa* mite loads. These random effect variables were then removed from the models due to the resultant lower AIC values when analyzing relationships between diseases and gene expression markers.

#### 2.7.1. Abiotic Stressor Analysis

Abiotic stressors (maximum daily temperature and air quality) in relationship with gene expression markers and diseases were accounted for in a multivariate Spearman rank correlation analysis.

#### 2.7.2. Biotic Stressor Analysis

The *N. ceranae* load was analyzed within foragers and in-hive bees in relation to the other numeric variables using a negative binomial generalized linear regression. For *N. ceranae* and *Varroa* mites, the date and apiary had significant random effects on the previous negative binomial regression analysis. Therefore, *N. ceranae* and *Varroa* mite loads were further analyzed across apiaries and time with Wilcoxon pairwise comparisons after applying relevant post hoc adjustments for multiple comparisons.

#### 2.7.3. Gene Expression Analysis

For each gene expression target (*Vg*, *ProPO*, and *HSP70*), the date had a significant random effect in the previous negative binomial regression analysis. Therefore, each gene target was further analyzed across time with Wilcoxon pairwise comparisons.

#### 2.7.4. Colony Detailed Assessment Correlation Analysis

Lastly, a multivariate Spearman rank correlation analysis was performed to determine the association between the detailed assessment measurements (FOB, number of adult bees, amounts of honey, nectar, pollen, and empty hive space), environmental measures (air quality index, and maximum daily temperature), the disease and pest loads (*N. ceranae* and *Varroa* mites), and gene expression measures (*ProPO*, *HSP70*, and *Vg*). 

#### 2.7.5. Histological Analyses of Bees

For the quantitative analysis of the histologically determined surface area of hypopharyngeal glands and mandibular glands, Student’s *t*-tests were performed for each comparison group using Prism 9 (GraphPad, v 9.4.1). 

## 3. Results

### 3.1. Abiotic Stressors—Temperature and Air Quality Index (AQI)

**Figure 2:**
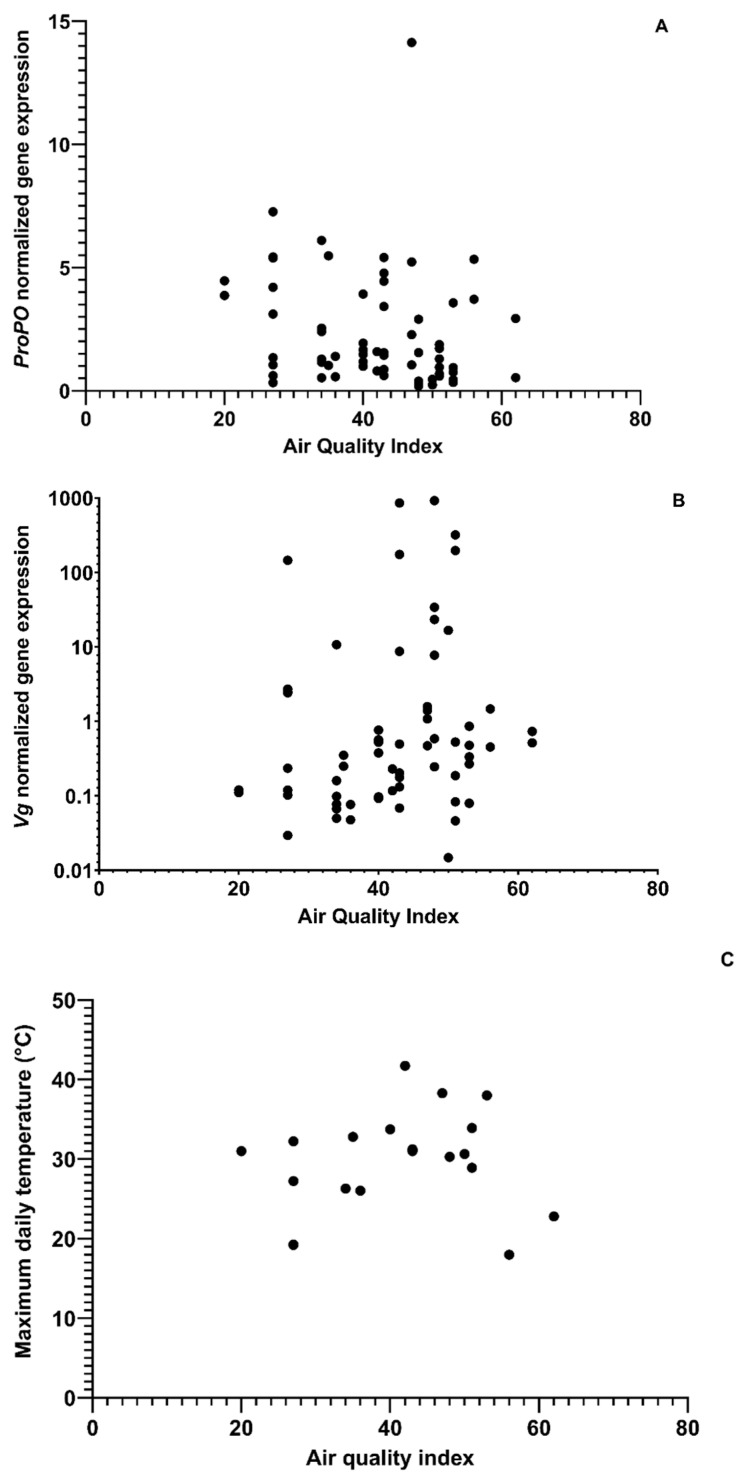
The correlation between air quality, (**A**) *Prophenoloxidase* (*ProPO*) normalized gene expression (ρ = −0.2839, df = 63, *p* = 0.023), (**B**) *Vitellogenin* (*Vg*) normalized gene expression (ρ = 0.26, df = 63, *p* = 0.038), and (**C**) Maximum daily temperature (ρ = 0.28, df = 63, *p* = 0.027).

The maximum daily temperature and AQI values were found to be positively correlated with each other (ρ = 0.28, df = 63, *p* = 0.027; Figure 2) and associated with the expression of *ProPO* and *Vg* genes (Figure 2). Forager bees that were exposed to poorer air quality had a negative association with *ProPO* gene expression (ρ = −0.2839, df = 63, *p* = 0.023; Figure 2A). Poor air quality was also positively correlated with the expression of the *Vg* gene (ρ = 0.26, df = 63, *p* = 0.038; Figure 2B). Among in-hive bees, there was a positive relationship between *HSP70* and *ProPO* gene expression (ρ = 0.60, df = 64, *p* < 0.001) (Table S2). Among foragers, there was a positive relationship between the expression levels of the *HSP70* gene and pollen stored in the hive. Adult bees were positively correlated with FOB and the maximum daily temperature (Table S3). Air quality index values varied more across apiaries in comparison to the maximum daily temperatures over the course of the study (Figure S1).

### 3.2. Biotic Stressors—N. ceranae and Varroa:

**Figure 3:**
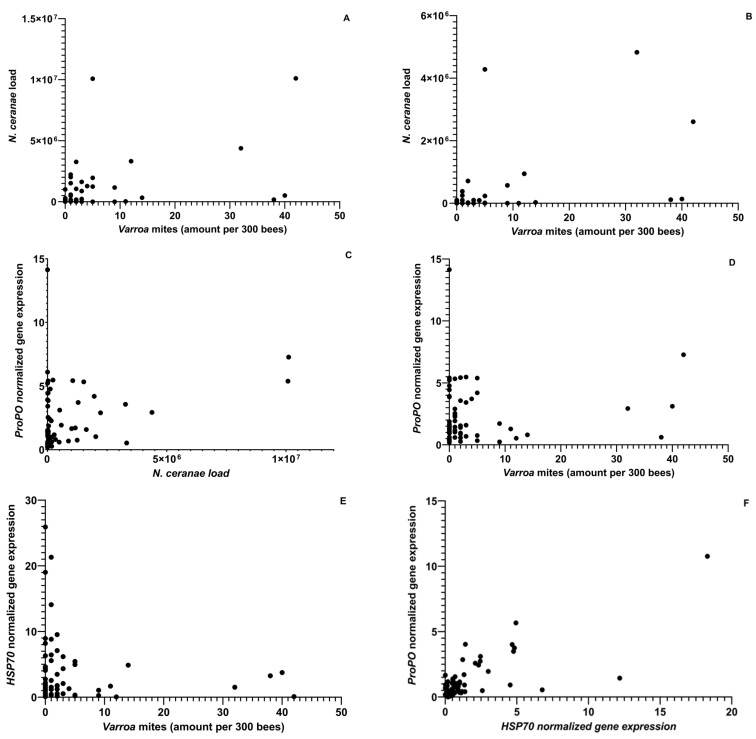
The correlation plots between (**A**) *N. ceranae* load in forager bees and colony *Varroa* mite loads (ρ = 0.51, df = 60, *p* < 0.0001), (**B**) *N. ceranae* loads in in-hive bees and colony *Varroa* mite loads (ρ = 0.43, df = 63, *p* = 0.004), (**C**) Normalized *Prophenoloxidase* (*ProPO*) gene expression and *N. ceranae* loads in forager bees (ρ = 0.27, df = 63, *p* = 0.03), (**D**) Normalized *Prophenoloxidase* (*ProPO*) gene expression in forager bees and colony *Varroa* mite loads (ρ = 0.0037, df = 60, *p* = 0.98), (**E**) *Heat Shock Protein 70* (*HSP70*) gene expression in forager bees and colony *Varroa* mite loads (ρ = −0.12, df = 60, *p* = 0.34) and (**F**) *Prophenoloxidase* (*ProPO*) gene expression in in-hive bees and *Heat Shock Protein 70* (*HSP70*) gene expression in in-hive bees (ρ = 0.60, df = 65, *p* < 0.0001).

**Figure 4:**
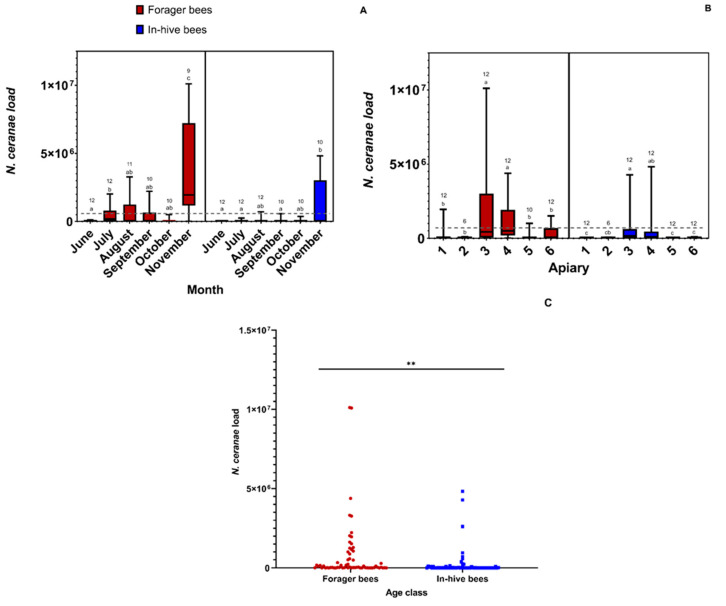
A box plot representing the *N. ceranae* load per colony found across (**A**) time and (**B**) Apiaries for both forager and in-hive bees. Medians are represented by solid lines within each box plot, and the boxes represent the inter-quartile range. The grey dashed line across the figure represents the threshold (1 million spores) at which a beekeeper is advised to treat their hives. The letters denote significant differences across months at the alpha = 0.05 level. The numbers above the bars represent sample sizes. (**C**) A dot plot representing the *N. ceranae* load in foragers and in-hive bees was pooled across all sampling sites and time points. The black horizontal line indicates which groups are being compared with the statistical test. The ** indicates a significant difference at *p* < 0.01.

**Figure 5:**
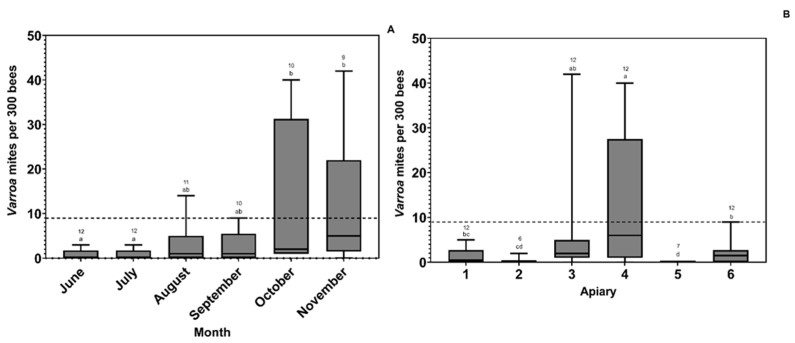
Box plots representing the number of *Varroa* mites per colony found across (**A**) Time and (**B**) Apiary sampled from the colony. Medians are represented by the solid lines within each box plot, and the boxes represent the inter-quartile range. The black dashed line across the figure represents the threshold (9 per 300 bees) at which a beekeeper is advised to treat their hive. The letters denote significant differences across the locations at the alpha = 0.05 level. The numbers above the letters represent the sample size.

The disease and pest incidences, *N. ceranae* and *Varroa* mites, were related to the gene expression levels of *ProPO* and *HSP70* (Figure 3). Loads of both stressors increased later in the season, with the highest levels recorded in the fall. Across foragers and in-hive bees, there was a positive relationship between the *Varroa* mite and *N. ceranae* loads (Negative binomial regression: χ^2^ = 4.98, df = 62, *p* = 0.026; χ^2^ = 5.34, df = 64, *p* = 0.021; Figure 3). The highest levels of *Varroa* and *N. ceranae* were found in two apiaries (3 and 4) that were near apiaries that did not receive any hive management measures to control these stressors (Figure 4 and Figure 5). 

#### 3.2.1. *N. ceranae* Analysis

The overall prevalence of *N. ceranae* infections across apiaries and all sampling dates was 72.31%. The pattern of *N. ceranae* loads in forager bees across the collection season is shown in Figure 4A, with loads in November appearing significantly higher than in the earlier months. The *N. ceranae* loads were the highest from sites 3 and 4 in comparison to the other apiary locations (Figure 4B). For in-hive bees, there was a similar trend; the highest loads were found in November in comparison to the other sampling months, and the highest levels of *N. ceranae* were found in sites 3 and 4 (Figure 4A,B). There were also significantly higher levels of *N. ceranae* in forager bees versus in-hive bees (negative binomial regression: χ^2^_1,129_ = 7.53, *p* = 0.0061; Figure 4C). 

#### 3.2.2. Varroa Mite Analysis

The overall prevalence of *Varroa* mite infestations across apiaries and all sampling dates was 60.00%. There were significantly higher *Varroa* mite loads in October and November in comparison to June. Across apiaries, there were significantly high *Varroa* mite loads in sites 3 and 4 in comparison to the rest (Figure 5). 

### 3.3. Gene Expression Analysis

For in-hive and forager bees, there were higher levels of the *HSP70* gene expression during the summer months. The highest *Vg* gene expression was in June and September for in-hive and forager bees. There were significantly lower levels of *ProPO* gene expression in September than in October and November for foragers, but in-hive bees showed higher levels in June than in the other months (Figure S2). Lastly, there were no significant differences in gene expression across apiaries for both foragers and in-hive bees. *N. ceranae* loads in forager bees were positively associated with *ProPO* gene expression (*N. ceranae:* ρ = 0.27, df = 63, *p* = 0.03). *Varroa* mite loads were positively associated with *ProPO* gene expression within forager bees (Negative binomial regression: χ^2^ = 6.50, df = 62, *p* = 0.011). However, there was a negative relationship between *Varroa* mite loads and forager bee *HSP70* gene expression (χ^2^ = 4.53, df = 62, *p* = 0.033; Figure 3). Among foragers, there was a positive relationship between *HSP70* gene expression and the amount of pollen stored in the hive. Among in-hive bees, there was a positive relationship between *HSP70* and *ProPO* gene expression (ρ = 0.60, df = 64, *p* < 0.001; Figure 3). 

### 3.4. Colony Detailed Assessment Correlation Analysis

Adult bees, FOB, brood, honey, and nectar stores (uncapped honey) were all positively correlated with one another and negatively correlated with empty space in the hive. Pollen was positively correlated with adult bees, the frames of bees, and brood. *N. ceranae* loads in forager bees were positively correlated only with nectar stores and the number of adult bees in the hive, while it was negatively correlated with the amount of empty space in the hive. There was also a positive correlation between the amount of honey and *Varroa* mite loads. There was a negative association between the maximum daily temperature and *Varroa* mites. All correlations and individual statistical values can be found in Table S3. 

### 3.5. Histological Analyses of Bees

**Figure 6:**
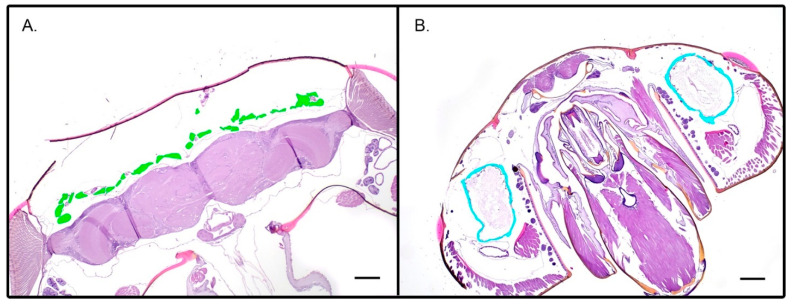
Representative histologic sections stained with Hematoxylin and eosin (H&E) stain through the head the for total area of hypopharyngeal glands (**A**) and mandibular glands (**B**). Image analysis. The highlighted areas indicate the total area measured. Scale bars represent 100 µM.

**Figure 7:**
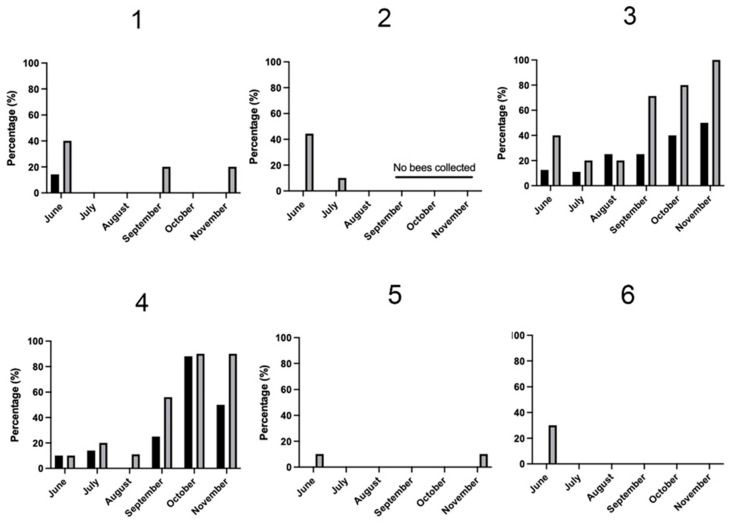
Percentage of *Nosema* spp. infection based on histologic evaluation over June–November 2021. The number 1–6 refer to the six different locations in the Napa, Yolo, and Solano counties of central California shown in Figure 1. Percent infection is based on ten nurse bees (black bars) and ten forager bees (gray bars) for each site and at each time point.

**Figure 8:**
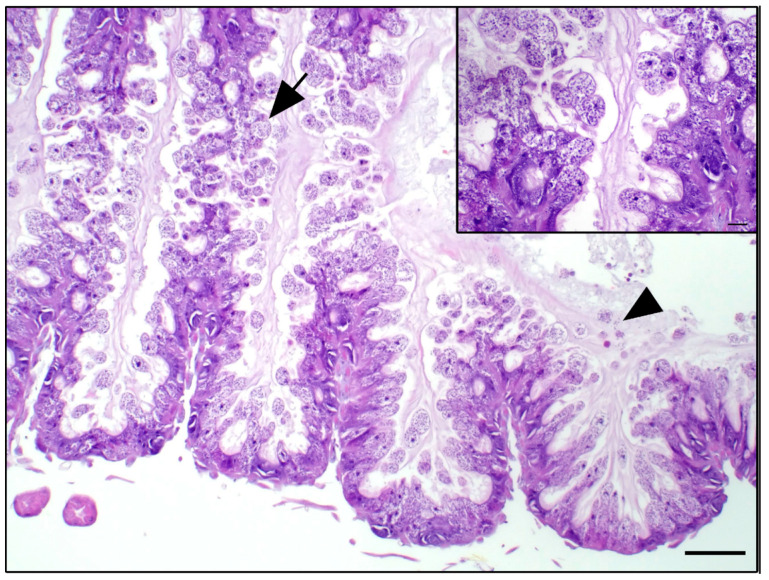
Representative microscopic images of *Nosema spp.* and sections of ventriculus in which epithelial cells were distended with abundant spores (arrow) and ventricular luminal spores (arrowhead), stained with Hematoxylin and eosin (H&E) stain. The scale bar represents 50 µM, and the inset scale bar represents 10 µM.

Based on the results of molecular findings and our interest in the effects of air quality and temperature on honey bee health and stress, the two hives with the greatest differences molecularly were histologically assessed for pathologic lesions. No trends or lesions were identified to correlate with air quality or temperature. Similarly, based on the results of the molecular findings, bees from two hives with the largest difference in gene expression, pest and disease loads, and climate variables were measured for their hypopharyngeal and mandibular gland size across each category (e.g., AQI, temperature, *Varroa* and *N. ceranae* loads, levels *ProPO, Vg, HSP70*). No trends or significant differences in gland sizes were identified for any group comparison (e.g., Figure 6). Finally, Nosemosis incidence was identified histologically and showed trends toward a high incidence in the October and November samples for two of the sites, while in the remaining sites, the pathogen was low or absent (Figure 7 and Figure 8). 

## 4. Discussion

Our results demonstrate relationships between climate change-mediated abiotic stressors (daily maximum temperature and air quality) on honeybee health parameters, including common biotic stressors—*N. ceranae* and *Varroa* mites—and the expression of different biomarker genes that are indicators of honeybee health. The positive relationship between daily maximum temperatures and poor air quality could lead to an increase in the susceptibility of forager bees to most likely be infected by a compromised immune system. Here, we found an increase in *ProPO* gene expression associated with high disease loads, yet with poorer air quality, there was an associated decrease in the *ProPO* gene expression. This enzyme is activated as part of a general innate immune response [50]; therefore, if its expression cannot be upregulated due to poor air quality, then it results in increased susceptibility of bees to particular diseases, such as viruses transmitted by *Varroa* mites. This relationship has been reiterated by a high correlation between the *ProPO* gene expression and Phenoloxidase activity across the development and castes of honeybees infested with *Varroa* mites [35]. In general, ProPO enzyme activity serves as one of the first lines of defense against diseases [50] in honeybees, and, in our study, this line of defense appears to be compromised with exposure to poor air quality.

Furthermore, *Varroa* mites were found to be correlated with a potentially lowered ability of foragers to buffer against high temperatures. The lowered expression of *HSP70* genes in our analysis suggests exacerbated challenges for honeybees if atmospheric temperatures continue to move toward the extremes in the future. Heat shock proteins were highly conserved across organisms and upregulated in response to temperature and other stressors. These HSP proteins chaperone the assembly, disassembly, folding, and unfolding of protein complexes so that their function can be preserved under stress [51]. The role of these proteins could become more critical as predicted temperature extremes become a reality in the future. While it is likely that ample pollen stores, as in our study hives, may facilitate an increase in the expression of *HSP70* gene expression, temperature stress, when combined with drought, may reduce pollen production in plants, thus limiting pollen availability for foraging bees [52,53]. Thus, the supplemental feeding of colonies could become critical for bees to retain their ability to combat abiotic stressors with appropriate responses at the physiological level, and this adds costs to hive management and increases the risks of hive losses.

Our results on the colony performance metrics indicate that the presence of *N. ceranae* or *Varroa* mites is not necessarily associated with a weak colony but instead may be dependent on other factors related to abiotic parameters, implying that colony-level indicators might not always be reliable. These discrepancies could relate to the differences in the seasonality of *N. ceranae* infections, where spore loads peak in the spring and are lowest in the fall [54], but whether these seasonal variations are linked to air temperatures is not fully understood. In our study, we did not measure *N. ceranae* loads in the spring, but we detected the highest levels in the fall and relatively stable levels throughout the rest of the beekeeping season, suggesting a chronic colony-level infection with this pathogen. The constant higher temperatures and poorer air quality year-round in the Central Valley of California during the study years might suppress the immune system of honeybees, as indicated by the lowering of the *ProPO* gene expression. This lowered expression could explain the higher disease loads observed year-round in this climate in comparison to more temperate climates, where *N. ceranae* levels are more seasonal [54]. The seasonal pattern we documented for *N. ceranae* here is similar to that reported in Spain, where *N. ceranae* is known to be particularly virulent [55]. Spain is a location with a Mediterranean climate like our study region, and *N. ceranae* infections in Spain are found year-round in colonies with a lack of seasonality for this pathogen [56]. Thus, our findings on disease loads and their relation to high daily temperatures and poor air quality have implications for other regions around the world that also have prevalent beekeeping operations and are experiencing climate change-related stressors resulting in drier and warmer weather patterns, as in our study area [57]. 

The higher amount of *N. ceranae* in forager bees versus in-hive bees is also supported by previous findings [58]. This difference is interesting to note, however, because forager bees are also subjected to more extreme climate conditions while foraging [59]. It is of interest to note that we did not detect *N. apis* in any of the samples based on the melt curve analysis, supporting the notion that *N. ceranae* is displacing *N. apis* throughout the world [60]. Our results also showed that poor air quality is associated with a decrease in *ProPO* gene expression in forager bees, suggesting that these bees, in an unfavorable environment, may have a decreased ability to combat infections and mount a strong immune response. Poor air quality showed a positive relationship with *Vg* expression in forager bees and increased *Vg* through upregulations, which is known to protect workers from oxidative stress, suggesting that poor air quality further stresses honeybee foragers. However, this trend does not hold in nurse bees which may be because they typically already have high levels of *Vg* expression [61]. The nurse bees only exhibit a positive correlation between *ProPO* and *HSP70* gene expression and not with any of the other stressors. These physiological changes in forager bees from poor air quality exposure may be further exacerbated by maximum daily temperatures, which have been shown to have a strong positive association with poor air quality [62]. Our results show associations between abiotic stressors and gene expression and lend support to the increase in challenges that bees are likely to face if there is a further decrease in air quality in this region especially relating to smoke from wildfires. 

Despite evidence that *Varroa* is known to suppress the immune system in newly emerged bees [35], we found a positive relationship with *ProPO* gene expression in forager bees. *Varroa* mites and *N. ceranae* were also positively correlated in this study. Therefore, this and other diseases present in *Varroa*-infested hives may likely cause a net increase in the innate immune response of forager bees. Supporting this notion, *Varroa* is known to vector viruses such as the Deformed Wing Virus (DWV) [33], which by itself has been shown to increase *ProPO* expression [63]. Therefore, there appears to be a net increase in the expression of the immune response *ProPO* gene in forager bees with a heavy disease burden. Thus, *ProPO* could serve as a dependable immune marker gene that reflects the ability of bees to fight and fend off potential infections.

Climate change for our study region is projected to lead to sustained increases in maximum daily temperatures in the future [2], which is associated with poorer air quality. Thus, with *Varroa* mites being negatively correlated with *HSP70* gene expression in forager bees, there is a strong likelihood of further hampering the ability of bees to tolerate extreme temperatures [34]. Furthermore, larvae without *Varroa* mites were found to have significantly lower *HSP70* gene expression levels [64]; however, in pupae, *Varroa* mite infestation caused a decrease in *HSP70* gene expression [65]. *Varroa* is known to vector virulent viruses, including fungal diseases such as stone brood, feed on fat body and hemolymph, as well as suppress the immune system [32,33,48,66] and, thus, is a major factor in the decline of bee health. Ongoing changes to climate could further augment the stresses caused by this parasitic mite. A closer look at our result showing a negative relationship between high temperatures and the *Varroa* mite loads in hives is indicative of some cautious optimism that the higher temperatures predicted in the future for this region might have benefits, as previous research demonstrated that *Varroa* mites were indeed sensitive to higher temperatures [67]. Bee heaters have been proposed as an effective method for killing and managing *Varroa* mite loads. However, this comes with several caveats, depending on the levels of high temperatures that honeybees can tolerate. However, it can be noted here that there are several heat shock proteins involved in proteostasis, and our study did not measure the capacity of bees to handle heat stress. Bees are known to have a slightly higher tolerance to high temperatures compared to *Varroa* mites, but it is also likely that this small beneficial impact could become minimal over an extended time as the parasite adapts to changing environmental conditions [68]. 

Histological analyses are gradually regaining center stage in research related to honeybee health, but our analyses show that histologic imaging may not be reliable to measure the impact of abiotic stressors on honeybees. While the pathological damage from *N. ceranae* infections is dependable and the loads detected followed similar trends to the molecular quantification analysis, the relationships between high temperature and poor air quality with expression levels of stress genes were weak. These abiotic stressors did not result in immediate signs of pathological damage to hypopharyngeal or mandibular glands that are indicators of honeybee health [42]. 

## 5. Conclusions

In summary, poor air quality is a serious environmental stressor impacting honeybees [24]. Air pollutants mask the floral odor receptors of honeybee antennae [25], limiting foraging success and colony nutrition. Our results demonstrate the relationship between air quality and daily maximum temperatures together can compound the impact of other stressors and the ability of bees to buffer against challenges. 

## Data Availability

The data collected corresponding to this project have been made publicly available through the open science framework and can be found at DOI 10.17605/OSF.IO/MC73D.

## Supplementary Materials

The following supporting information can be downloaded at: https://www.mdpi.com/article/10.3390/insects14080689/s1, Figure S1: The maximum daily temperatures measured at the closest weather station to each apiary and the corresponding air quality index value from each weather station; Figure S2: A box plot representation of the medians and inter-quartile ranges of expression levels of (A) Heat Shock Protein (HSP70), (B) Vitellogenin (Vg), and (C) Prophenoloxidase (ProPO) genes across the months the hives were sampled for forager and in-hive bees. Table S1: Primers for *Nosema* quantification as well as for each gene expression target.; Table S2: Spearman rank correlation values showing the relationship between maximum air temperatures and Air Quality Index (AQI) values. Table S3: The statistical results from a multivariate spearman rank analysis between Variables X and Y.
