# Supplementary material for: Poor Air Quality Is Linked to Stress in Honeybees and Can Be Compounded by the Presence of Disease
Source: Insects. 2023 Aug 4;14(8):689. doi: 10.3390/insects14080689 (PMC10455886; doi:10.3390/insects14080689)
Supplement: Supplementary file 1 [file insects-14-00689-s001.zip › insects-2434195-supplementary.pdf]

Supplemental Information

Table S1. Primers for *Nosema* quantification as well as for each gene expression target. The oligonucleotide sequence is provided along with the product length and a reference source for each primer set.

**Target**	**Primer name**	**Primer Sequence**	**Product Length (bp)**	**Reference**
Nosema apis	F-NAPIS	GGGGGCATGTCTTTGACGTACTATGTA	321	[1]
	R-NAPIS	GGGGGGCGTTTAAAATGTGAAACAACTATG		
Nosema ceranae	F-NCERANAE	CGGCGACGATGTGATATGAAAATATTAA	218	[1]
	R-NCERANAE	CCCGGTCATTCTCAAACAAAAAACCG		
Heat Shock Protein 70 (*HSP70*)	F-HSP70	CGCCTTCACGGACACAGA	60	[2]
	R-HSP70	TTCATTGCGACCTGATTTTTG		
Vitellogenin (*Vg*)	Vg-F	CTGTCGATGGAGAAGGGAACT	370	[3]
	Vg-R	CTTGCCTACGAGTCTTGCTGT		
Prophenoloxidase (*ProPO*)	F-ProPO	ACAGATCCTGTATGGATTGC	61	[4]
	R-ProPO	TCTTGGACGAGTAAAACGAT		
Ribosomal Protein S5 (RpS5) (reference gene)	RpS5-F	AATTATTTGGTCGCTGGAATTG	115	[3]
	RpS5-R	TAACGTCCAGCAGAATGTGGTA		

Table S2. Spearman rank correlation values showing the relationship between maximum air temperatures and Air Quality Index (AQI) values. * and **Bold** indicate significant differences at the alpha = 0.05 level. *ProPO*: Prophenoloxidase gene; *Vg*: Vitellogenin gene; *HSP70*: Heat Shock Protein 70 gene

	Maximum Temperature	*Varroa*	*N. ceranae*	*ProPO*	*Vg*	*HSP70*
AQI	**0.277***	0.029	0.078	**-0.284***	**0.260***	-0.244
Maximum Temperature		-0.336	-0.146	-0.093	-0.007	0.204

Figure S1. (A) The maximum daily temperatures measured at the closest weather station to each apiary. (B) The corresponding air quality index value from each weather station closest to the apiary sampled. Each dot represents a different apiary location. The lines represent the mean of the maximum daily temperature or air quality value.

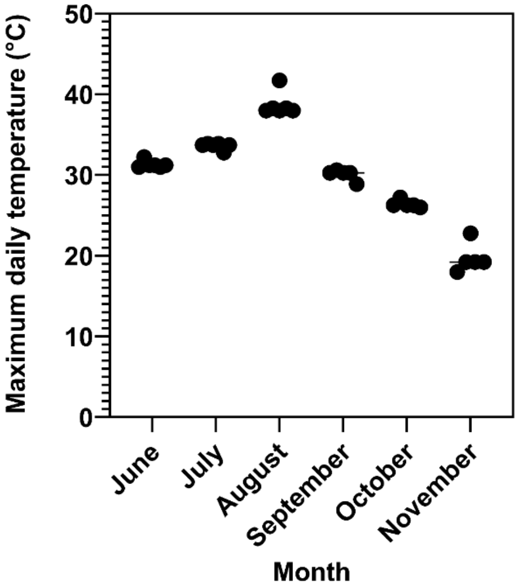

A

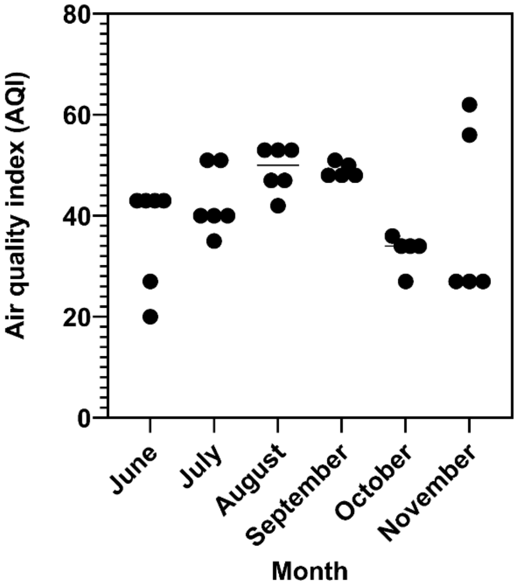

B

Figure S2. A box plot representation of the medians and inter-quartile ranges of expression levels of (A) *Heat Shock Protein* (*HSP70*), (B) *Vitellogenin* (*Vg*), and (C) *Prophenoloxidase* (*ProPO*) genes across the months the hives were sampled for forager and in-hive bees. The letters above each box plot represent significant differences at the alpha = 0.05 level. The numbers above each letter represent the sample size of the hives sampled for each month.

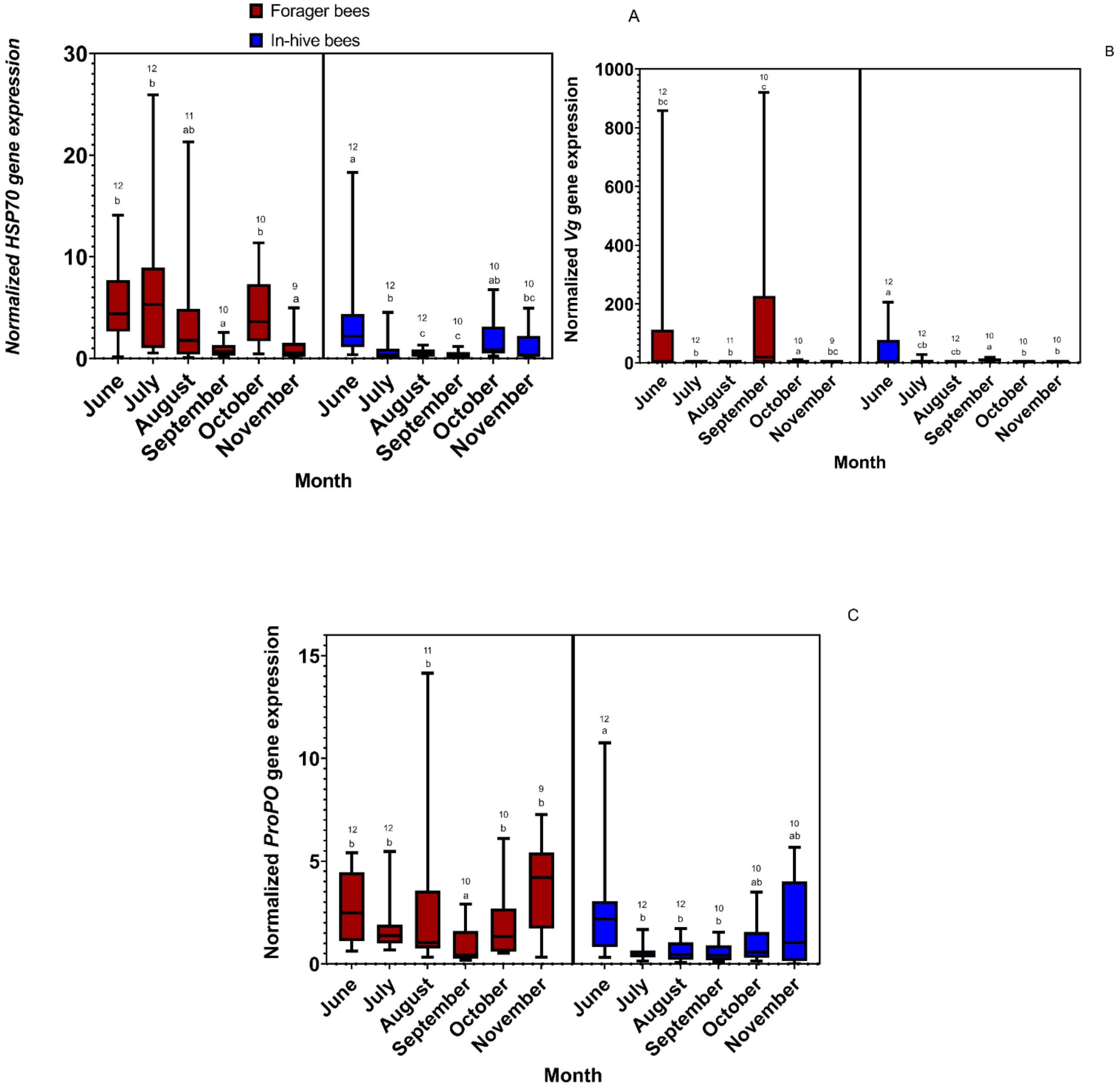

Table S3. The statistical results from a multivariate spearman rank analysis between Variables X and Y. The last column visualizes the strength of the relationship between the two variables corresponding to the spearman rank ρ value. The * indicates a significant relationship between the two variables at the alpha = 0.05 level. *ProPO*: *Prophenoloxidase* gene; *Vg*: *Vitellogenin* gene; *HSP70*: *Heat Shock Protein 70* gene; FOB: Frames of Bees; AQI: Air Quality Index;

**Variable X**	**Variable Y**	**Spearman ρ**	**P-value**	**Strength of ρ value**
HSP70	Nosema	-0.0044	0.9724	
Vg	Nosema	0.0702	0.5813	
Vg	HSP70	-0.1605	0.2052	
ProPO	Nosema	0.2715	0.0300*	
ProPO	HSP70	0.2993	0.0163*	
ProPO	Vg	0.2369	0.0595	
AQI	Nosema	0.0781	0.5398	
AQI	HSP70	-0.2438	0.0523	
AQI	Vg	0.2600	0.0380*	
AQI	ProPO	-0.2839	0.0230*	
Max_Temp	Nosema	-0.1463	0.2486	
Max_Temp	HSP70	0.2041	0.1058	
Max_Temp	Vg	-0.0073	0.9542	
Max_Temp	ProPO	-0.0930	0.4651	
Max_Temp	AQI	0.2766	0.0270*	
FOB	Nosema	0.2001	0.1129	
FOB	HSP70	0.0096	0.9399	
FOB	Vg	0.0896	0.4815	
FOB	ProPO	-0.0768	0.5461	
FOB	AQI	0.1446	0.2543	
FOB	Max_Temp	0.4927	<.0001*	
Varroa mites	Nosema	0.5148	<.0001*	
Varroa mites	HSP70	-0.1247	0.3385	
Varroa mites	Vg	-0.2197	0.0888	
Varroa mites	ProPO	0.0037	0.9773	
Varroa mites	AQI	0.0289	0.8252	
Varroa mites	Max_Temp	-0.3360	0.0081*	
Varroa mites	FOB	-0.0373	0.7756	
Adult_Bees	Nosema	0.4004	0.0385*	
Adult_Bees	HSP70	0.0965	0.6321	
Adult_Bees	Vg	-0.0009	0.9964	

Adult_Bees	ProPO	0.1884	0.3467	
Adult_Bees	AQI	-0.0703	0.7275	
Adult_Bees	Max_Temp	0.3070	0.1193	
Adult_Bees	FOB	0.9403	<.0001*	
Adult_Bees	Varroa mites	0.0997	0.6354	
Brood	Nosema	0.3732	0.0552	
Brood	HSP70	0.2169	0.2772	
Brood	Vg	-0.1808	0.3667	
Brood	ProPO	0.3131	0.1118	
Brood	AQI	-0.1623	0.4185	
Brood	Max_Temp	0.2880	0.1452	
Brood	FOB	0.8234	<.0001*	
Brood	Varroa mites	0.1467	0.4842	
Brood	Adult_Bees	0.8739	<.0001*	
Honey	Nosema	0.2759	0.1636	
Honey	HSP70	-0.0879	0.6627	
Honey	Vg	-0.1146	0.5693	
Honey	ProPO	0.0619	0.7591	
Honey	AQI	0.1637	0.4144	
Honey	Max_Temp	-0.1015	0.6144	
Honey	FOB	0.5491	0.0030*	
Honey	Mites	0.5759	0.0026*	
Honey	Adult_Bees	0.4189	0.0296*	
Honey	Brood	0.3295	0.0933	
Nectar	Nosema	0.4780	0.0117*	
Nectar	HSP70	0.0530	0.7928	
Nectar	Vg	-0.1980	0.3222	
Nectar	ProPO	0.0898	0.6560	
Nectar	AQI	-0.1276	0.5260	
Nectar	Max_Temp	-0.3106	0.1148	
Nectar	FOB	0.6007	0.0009*	
Nectar	Varroa mites	0.3737	0.0657	
Nectar	Adult_Bees	0.4339	0.0237*	
Nectar	Brood	0.3851	0.0473*	
Nectar	Honey	0.6315	0.0004*	
Pollen	Nosema	0.2858	0.1484	
Pollen	HSP70	0.4075	0.0349*	

Pollen	Vg	0.1634	0.4154	
Pollen	ProPO	0.2419	0.2241	
Pollen	AQI	-0.2920	0.1395	
Pollen	Max_Temp	0.2195	0.2713	
Pollen	FOB	0.5338	0.0041*	
Pollen	Varroa mites	-0.2024	0.3319	
Pollen	Adult_Bees	0.6517	0.0002*	
Pollen	Brood	0.6620	0.0002*	
Pollen	Honey	-0.1480	0.4612	
Pollen	Nectar	0.1116	0.5795	
Empty	Nosema	-0.4749	0.0123*	
Empty	HSP70	-0.0880	0.6626	
Empty	Vg	0.2144	0.2828	
Empty	ProPO	-0.1283	0.5236	
Empty	AQI	-0.0203	0.9201	
Empty	Max_Temp	-0.1038	0.6063	
Empty	FOB	-0.8949	<.0001*	
Empty	Varroa mites	-0.4336	0.0304*	
Empty	Adult_Bees	-0.8458	<.0001*	
Empty	Brood	-0.7880	<.0001*	
Empty	Honey	-0.7037	<.0001*	
Empty	Nectar	-0.6997	<.0001*	
Empty	Pollen	-0.3946	0.0417*	

References

1. Burgher-MacLellan, K.L.; Williams, G.R.; Shutler, D.; MacKenzie, K.; Rogers, R.E.L. Optimization of Duplex Real-Time PCR with Meltingcurve Analysis for Detecting the Microsporidian Parasites *Nosema apis* and *Nosema ceranae* in *Apis mellifera*. *The Canadian Entomologist* **2010**, *142*, 271-283, 213.
2. Morammazi, S.; Shokrollahi, B. The pattern of HSP70 gene expression, flight activity and temperature in Apis mellifera meda colonies. *J Therm Biol* **2020**, *91*, 102647, doi:10.1016/j.jtherbio.2020.102647.
3. Hamiduzzaman, M.M.; Emsen, B.; Hunt, G.J.; Subramanyam, S.; Williams, C.E.; Tsuruda, J.M.; Guzman-Novoa, E. Differential Gene Expression Associated with Honey Bee Grooming Behavior in Response to Varroa Mites. *Behav Genet* **2017**, *47*, 335-344, doi:10.1007/s10519-017-9834-6.
4. Zaobidna, E.A.; Żółtowska, K.; Łopieńska-Biernat, E. Expression of the Prophenoloxidase Gene and Phenoloxidase Activity, During the Development of Apis Mellifera Brood Infected with Varroa Destructor. J. Apic. Sci. **2015**, *59*, 85-93, doi:10.1515/jas-2015-0025.
